# Daily Bisphenol A Excretion and Associations with Sex Hormone Concentrations: Results from the InCHIANTI Adult Population Study

**DOI:** 10.1289/ehp.1002367

**Published:** 2010-08-25

**Authors:** Tamara Galloway, Riccardo Cipelli, Jack Guralnik, Luigi Ferrucci, Stefania Bandinelli, Anna Maria Corsi, Cathryn Money, Paul McCormack, David Melzer

**Affiliations:** 1 School of Biosciences, University of Exeter, Exeter, United Kingdom; 2 European Centre for Environment and Human Health, Peninsula College of Medicine and Dentistry (PCMD), Truro, United Kingdom; 3 Laboratory of Epidemiology, Demography, and Biometry and; 4 Clinical Research Branch, National Institute on Aging, National Institutes of Health, Department of Health and Human Services, Baltimore, Maryland, USA; 5 InCHIANTI Group, Piero Palagi Hospital, Florence, Italy; 6 Brixham Environmental Laboratory, AstraZeneca UK Ltd., Devon, United Kingdom; 7 Epidemiology and Public Health, Peninsula College of Medicine and Dentistry, University of Exeter, Exeter, United Kingdom

**Keywords:** endocrine disruption, androgen, antiandrogen, bisphenol A, human biomonitoring, health effects, InCHIANTI

## Abstract

**Background:**

Bisphenol A (BPA) is a high production volume chemical widely used in packaging for food and beverages. Numerous studies have demonstrated that BPA can alter endocrine function in animals, yet human studies remain limited.

**Objective:**

We estimated daily excretion of BPA among adults and examined hypothesized associations with serum estrogen and testosterone concentrations.

**Methods:**

We conducted cross-sectional analyses using data from the InCHIANTI Study, a prospective population-based study of Italian adults. Our study included 715 adults between 20 and 74 years old. BPA concentrations were measured by liquid chromatography–mass spectrometry in 24-hr urine samples. The main outcome measures were serum concentrations of total testosterone and 17β-estradiol.

**Results:**

Geometric mean urinary BPA concentration was 3.59 ng/mL [95% confidence interval (CI), 3.42–3.77 ng/mL], and mean excretion was 5.63 μg/day (5th population percentile, 2.1 μg/day; 95th percentile, 16.4 μg/day). We found higher excretion rates among men, younger respondents, and those with increasing waist circumference (*p* = 0.013) and weight (*p* = 0.003). Higher daily BPA excretion was associated with higher total testosterone concentrations in men, in models adjusted for age and study site (*p* = 0.044), and in models additionally adjusted for smoking, measures of obesity, and urinary creatinine concentrations (β = 0.046; 95% CI, 0.015–0.076; *p* = 0.004). We found no associations with the other serum measures. We also found no associations with the primary outcomes among women, but we did find an association between BPA and SHBG concentrations in the 60 premenopausal women.

**Conclusion:**

Higher BPA exposure may be associated with endocrine changes in men. The mechanisms involved in the observed cross-sectional association with total testosterone concentrations need to be clarified.

Bisphenol A (BPA) is a synthetic compound that is a suspected endocrine disruptor—a compound capable of causing dysfunction to hormonally regulated body systems (Talsness et al. 2009). BPA is used as a monomer in polycarbonate plastics and in the epoxy resins that are used to line food and beverage containers; it is one of the world’s highest production volume chemicals (Burridge 2003). Widespread and continuous daily exposure to BPA is believed to occur primarily through the diet (Stahlhut et al. 2009), as well as from drinking water, dental sealants, dermal exposure, and inhalation of household dusts. The presence of measurable concentrations of metabolites has been reported in the urine of > 90% of people in population-representative samples from across the globe (Calafat et al. 2008; Vandenberg 2007).

Most studies of the health effects of BPA have focused on its well-documented estrogenic activity, with reports of both estrogen agonist (Lee et al. 2003) and androgen antagonist activity (Bonefeld-Jørgensen et al. 2007; Lee et al. 2003; Okada et al. 2008). Suppression of aromatase activity has been observed in laboratory studies (Bonefeld-Jørgensen et al. 2007), as has binding to alternative nuclear receptors, including the aryl hydrocarbon receptor (Kruger et al. 2008) and estrogen-related receptor γ, the function of which remains unknown (Okada et al. 2008). In addition, BPA has been reported to cause thyroid hormone disruption (Moriyama et al. 2002), altered pancreatic beta-cell function (Ropero et al. 2008), and obesity-promoting effects (Newbold et al. 2008). The potential for low-dose effects has prompted debate on revising the current legislation of recommended safe daily exposure levels (Beronius et al. 2010; vom Saal et al. 2007).

Based on the animal and laboratory evidence, we previously hypothesized that higher urinary BPA concentrations would be associated with adverse human health effects. Using data from the U.S. National Health and Nutrition Examination Survey (NHANES) for 2003–2004, the first large-scale population-based epidemiological data on urinary BPA concentrations with sufficient power to detect low-dose effects, we showed for the first time a clear correlation between BPA exposure and disease in humans (Lang et al. 2008). Higher BPA concentrations in NHANES respondents were associated with diagnoses of cardiovascular disease and diabetes but not with other common diseases, which suggested specificity of the reported findings (Melzer et al. 2008, 2009). We recently used an entirely new study sample from the 2005–2006 NHANES to conduct an independent replication of the association of BPA and cardiovascular disease (Melzer et al. 2010). The results of this replication indicated that chance was an implausible explanation for our results.

Studies to clarify the mechanisms of these associations are clearly a priority. A substantive literature documents the disruption of circulating reproductive hormone concentrations after BPA exposures in animal models (reviewed by Richter et al. 2007; see also Bonefeld-Jørgensen et al. 2007; Goodman et al. 2009; Talsness et al. 2009). Studies of human populations have until now been limited to very small sample sizes. A significant, positive relationship was reported between circulating androgen concentrations and BPA exposure in a small study of 26 normal women and 47 women with ovarian dysfunction (Takeuchi et al. 2004). More recently, Meeker et al. (2010) studied serum thyroid and reproductive hormone levels in 167 men recruited through an infertility clinic and observed inverse relationships between urinary BPA concentrations and the free androgen index [ratio of testosterone to sex hormone–binding globulin (SHBG)], estradiol, and thyroid-stimulating hormone. Given these findings, we hypothesized that higher urinary BPA concentrations would be associated with altered reproductive hormone concentrations in serum. Because a limitation of previous studies has been their reliance on single spot urine samples, we based our current analysis on 24-hr urine collections, to provide a direct measure of daily excretion rates. We selected participants from the InCHIANTI study (Aging in the Chianti Area, Tuscany, Italy), a representive population-based study that was conducted in Chianti, Italy, from September 1998 to March 2000. Our analysis of the data from this sample provides the first report of daily BPA excretion levels in a large European cohort.

## Materials and Methods

### Study population

The InCHIANTI study (InCHIANTI 2010) was designed to identify risk factors for mid- and late-life morbidity and has been described extensively elsewhere (Ferrucci et al. 2000).

Briefly, InCHIANTI is a prospective population-based study of a suburban and rural town population. City registries were used to randomly select adults who were living in Greve in Chianti and in Bagno a Ripoli, Tuscany, Italy; a multistage sampling method was used (296 adults < 65 years old, 533 adults 65–74 years old, and 102 adults ≥ 75 years old; response rate, 91.6% from baseline interview). In line with previous work, we have limited our analysis here to participants ≤ 74 years old. The Instituto Nazionale Riposo e Cura Anziani Institutional Review Board provided ethical approval for the study. Participants gave informed consent, or if they were unable to do so, a close relative provided surrogate consent.

### Analysis of urinary BPA concentrations

Analysis of samples was performed (under contract) at the Brixham Environmental Laboratory, (Brixham, UK) in compliance with Good Laboratory Practice. Because orally administered BPA is considered to be rapidly and completely excreted, urine is the body fluid most appropriate for the biomonitoring assessment of BPA exposure (see Calafat et al. 2005). To measure total (free and conjugated) urinary concentrations of BPA, we used the methods employed by NHANES (Calafat et al. 2008) and adopted by the Division of Environmental Health Laboratory Sciences, National Center for Environmental Health, Centers for Disease Control and Prevention to prepare the urine samples; we performed the analyses using online solid-phase extraction (SPE) coupled with high-performance liquid chromatography (LC)–isotope dilution tandem mass spectrometry (MS/MS) with peak focusing. Analyses where carried out using a commercially available, integrated online SPE-LC system (Symbiosis Pharma System; Spark Holland BV, Emmen, the Netherlands) coupled with a triple-quadrupole mass spectrometer equipped with a heated electrospray ionization (HESI) interface (TSQ Quantum Ultra AM; Thermo Scientific, Hemel Hempstead, UK). Two major advantages of the Symbiosis Pharma system are that a new SPE cartridge is used for every analysis and that one SPE cartridge is prepared while one is being analyzed. This system enabled a 7-min SPE-LC-HESI/MS/MS run time for each analysis point. A linear calibration was obtained from 0.50–100 μg/L (*R*^2^ > 0.996). The limit of detection (LOD) was < 0.50 μg/L BPA, the limit of quantitation was 0.50 μg/L BPA, the lowest calibration standard with a signal height: noise height ratio > 10 (relative standard deviations < ±20%, all other standards < ±15%).

### Outcomes

Participants who consented to donate a blood sample were asked also to collect the urine for 24 hr in a vessel containing 3 g boric acid as preservative. During the 3 days before blood and urine collection, the subjects consumed a diet free of meat and fish. On the morning of the day before the blood samples were drawn, participants urinated and flushed away the first voided urine and then began the urine collection. During the day and night, all the produced urine was saved into the plastic bottle stored at room temperature or in the refrigerator. After 24 hr, bottles were weighed and the total volume measured in the clinic.

First thing the next morning, after having been sedentary for 15 min, fasting blood samples were collected for routine blood examination. Aliquots of serum and plasma were subsequently prepared and stored at −80°C for additional analyses. A 24-hr urine sample aliquot (70 mL) was stored at −20°C until further analyses.

Testosterone that circulates in the blood binds predominantly to protein, with approximately 40% bound to the high-affinity SHBG and 60% to albumin with lower affinity. Measurement of serum testosterone typically includes estimating total testosterone (free plus bound), free testosterone (not protein bound), and bioavailable testosterone (not SHBG bound).

Total testosterone was assessed through a commercial radioimmunological assay (RIA) kit (Active Testosterone RIA DSL-4000; Diagnostic Systems Laboratories, Webster, TX, USA, distributed by Chematil, s.r.l., Angri SA, Angri, Italy). The minimum LOD was 0.08 ng/mL. Intraassay coefficients of variation (CVs) for three different concentrations ranged from 7.8–9.6%, and interassay CVs ranged from 8.4–9.1%. Results were transformed and reported as nanograms per milliliter according to the manufacturer’s instructions.

SHBG level was measured by RIA (IRMA DSL-7400; Diagnostic Products Corp., Los Angeles, CA, USA). The analytical sensitivity was 3 nmol/L. The intraassay CVs for three different concentrations were 1.1–3.7%, and interassay CVs were 8.7–11.5%.

Free testosterone was estimated from measured total testosterone, SHBG, and albumin (4.3 g/dL) using the method described by Vermeulen et al. (1999; for a worked example, see the International Society for the Study of the Aging Male 2010).

Estradiol levels were measured using an ultrasensitive RIA (Ultra-sensitive Estradiol RIA DSL-4800; Diagnostic Systems Laboratories, distributed by Chematil). The theoretical sensitivity was 2.2 pg/mL. Intraassay CVs across four different concentrations ranged from 6.5–8.9%, and interassay CVs ranged from 7.5–12.2% (at 108.7 pg/mL).

### Statistical analyses

Descriptive statistics of urinary BPA concentration and serum hormone levels were tabulated. We calculated geometric means and distribution percentiles of two different BPA measures. First, we measured the BPA volume concentration (expressed as micrograms of BPA/liter of urine). Then, we multiplied the BPA concentration by the urine collection rate (liters/day), which was measured considering the urine volume collected in 24 hr, and thus obtained the urinary excretion rate of BPA (expressed as micrograms/day).

We performed multivariate linear regression analyses to study the association between BPA and a broad range of demographic covariates and possible confounders. Because the concentrations of daily BPA excretion were not normally distributed, we used natural log transformation when BPA was considered the dependant variable. BPA values were not transformed when it was considered an explanatory variable in serum hormone examination. In all analyses, an upper age cutoff was 75 years to minimize the problem of comorbidity.

We adjusted our models by selecting different covariates. The variables included in our analyses were age, reported in years at the last birthday and used as a continuous variable; the two municipalities (study sites) where participants lived; waist circumference (centimeters) and weight (kilograms); and body mass index (BMI) was calculated as the weight (kilograms) divided by the square of height (meters). BMI was tested as a continuous variable and as a categorized dummy variable with subjects divided into underweight (< 18.5 kg/m^2^), recommended weight (18.5–24.9 kg/m^2^), overweight (25.0–29.9 kg/m^2^), obese I (30.0–34.9 kg/m^2^), and obese II (≥ 35 kg/m^2^) categories. We also considered smoking status, which appeared to be correlated to BPA in unadjusted models.

Urinary creatinine concentration is commonly used to adjust within-day variation in metabolite analysis from single spot urine samples (Barr et al. 2005). Linear regression analysis using the outcome (hormone) measures as the dependant variable was performed first considering all the subjects and then considering men and women separately. Data analysis was performed using STATA (version 10 SE; StataCorp LP, College Station, TX, USA); *p* < 0.05 was considered significant.

## Results

The geometric mean urinary concentration of BPA was 3.59 ng/mL [95% confidence interval (CI), 3.42–3.77 ng/mL; Table 1]. Based on the 24-hr urine collection, the daily excretion rate of BPA had a geometric mean of 5.63 μg/day but varied widely. The distribution was skewed, with a 10th percentile of 2.6 μg/day (95% CI, 2.5–2.8 μg/day) and a 90th percentile of 11.8 μg/day (95% CI, 10.9–12.7 μg/day). Daily BPA excretion was lower among women than amomg men (*p* < 0.001 in models adjusted for age, sex, and study site) and lower with advancing age (*p* < 0.001). We obtained identical results both with and without correction for creatinine. In models adjusted for age, sex, and study site (Table 2), we found no associations between daily BPA excretion and years of education or smoking status. We did find associations with waist circumference (β = 0.0062; 95% CI, 0.0016–0.0108; *p* = 0.013) and with weight (β = 0.0064; 95% CI, 0.0023–0.0104; *p* = 0.003).

In models for men, adjusted for age and study site, we found no association between BPA excretion and 17β-estradiol. However, we did find a significant association between daily BPA excretion and total testosterone concentration (β = 0.0237; 95% CI, 0.0006–0.0468; *p* = 0.044). In models adjusted for age, study site, smoking, BMI, weight, waist, and urinary creatinine, the BPA association with total testosterone levels was highly significant (β = 0.046; 95% CI, 0.015–0.076; *p* = 0.004; Table 3).

To explore further the association with testosterone in men, we examined associations with the derived measure of free testosterone, based on SHBG concentrations. The association between BPA excretion and free testosterone narrowly missed significance (*p* = 0.075 in fully adjusted models).

For women, the geometric mean of the 17β-estradiol concentration was 6.89 pg/mL (Table 4), but this varied dramatically by menopause status: 22.4 pg/mL (95% CI, 16.7–30.0 pg/mL) in the 57 premenopausal women and 5.3 pg/mL (95% CI, 4.8–5.7 pg/mL) in the 290 postmenopausal women. In the models that tested hormone associations with BPA excretion among women (Table 4), we found no significant associations for either estradiol or total testosterone. Both SHBG concentration and the derived measure of free testosterone showed significant associations with BPA excretion in premenopausal women, although it should be noted that the method used (direct measure of free testosterone by RIA and calculation of the free androgen index) was not designed for measuring androgen concentrations in women, where the concentrations involved are at the very lowest LODs (Miller et al. 2004; Vermeulen et al. 1999).

### Sensitivity analysis

**Figure 1:**
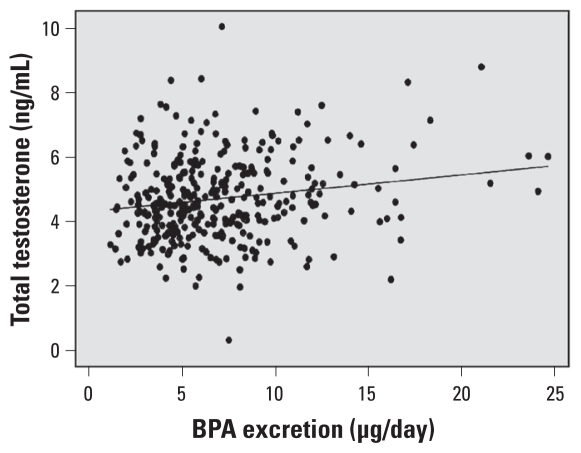
Scatter plot of BPA excretion per day against total testosterone concentrations, with unadjusted linear regression line (BPA outlier values censored at < 25 μg/day).

For a sensitivity analysis of our main finding, we examined the relationship between daily BPA excretion and total testosterone levels in men, excluding outlier BPA values above 25 μg/day (*n* = 7 removed, ranging from 25.29–41.12 μg/day) (see Figure 1 for unadjusted model). In fully adjusted models as above, BPA excretion per day remained associated with total testosterone concentrations in men (β = 0.0521; 95% CI, 0.0172–0.08703; *p* = 0.004).

Post hoc analyses for bioavailable testosterone showed patterns similar to those reported for free testosterone (data not shown). Associations with estradiol: testosterone ratios were nonsignificant.

## Discussion

In this study, we have reported for the first time the daily excretion levels of BPA among European adults in a large-scale and high-quality population-based sample. After adjusting for potential confounders, we have shown that higher BPA daily excretion was associated with an increase in serum total testosterone concentration in men.

These results are important because they provide the first report, using data from a large-scale human population, of associations between elevated exposure to BPA and alterations in circulating hormone levels. They also illustrate that the extent of exposure to BPA is similar in this European urban and rural population to exposures seen in the general adult population of the United States (Calafat et al. 2008). Previous studies of the relationship between human exposure to BPA and endocrine function are sparse and involve reported alterations in androgens (gonadotrophins or testosterone) in urine or serum in both men and women, although the numbers of participants were small (Hanaoka et al. 2002; Takeuchi and Tsutsumi 2002; Takeuchi et al. 2004). Hanaoka et al. (2002) studied 42 occupationally exposed male production workers and age-matched controls and showed that urinary BPA concentrations were inversely associated with follicle-stimulating hormone (FSH) but not with free testosterone or leutinizing hormone. In a later study of 167 men recruited through an infertility clinic (Thuillier et al. 2009), BPA concentrations in urine were positively associated with both FSH and FSH:inhibin ratio and inversely associated with estradiol:testosterone ratio. Because FSH and inhibin B are the two hormones considered most predictive of semen quality, Thuillier et al. (2009) concluded that BPA may have been associated with adverse effects on Sertoli cells or their FSH receptors that led to altered inhibin B production and reduced semen quality. In an animal study, rats exposed to BPA *in utero* did not show significant changes in circulating testosterone levels in adulthood, which suggests normal functioning of Leydig and Sertoli cells (Goodman et al. 2009). Because estrogens and androgens can exert differential effects in function depending on the cell type and its stage of development, the consequences of BPA exposure on adult reproductive and somatic tissues merits further attention.

Our results showed an association with total testosterone concentrations but no significant trend in 17β-estradiol levels with higher BPA excretion in men. The results reported by Meeker et al. (2010) are consistent with those reported here, although the positive trend (*p* = 0.17) between BPA and testosterone reported by Meeker et al. (2010) did not reach statistical significance in their smaller study. Mendiola et al. (2010) reported finding no association between urinary BPA concentrations and testosterone levels in 375 male partners of pregnant women; in addition to differences in study group, their urinary BPA concentrations appear substantially lower than in our study sample.

Plausible explanations for our finding of an increase in total testosterone include a reduction in aromatase activity (Akingbemi et al. 2004; Huang and Leung 2009; Nativelle-Serpentini et al. 2003), which would lead to a decrease in the conversion of testosterone to estradiol. Because BPA has been shown to possess antiandrogenic activity (Bonefeld-Jørgensen et al. 2007; Lee et al. 2003), an alternative explanation could be that a blockade of androgen-binding sites alters feedback control mechanisms that leads to an increase in circulating testosterone. Lee et al. (2003) showed BPA to affect multiple steps in the activation and function of the androgen receptor, including noncompetitive inhibition of binding of endogenous androgens, nuclear localization, and transactivation, with uncertain consequences for androgen homeostasis. In our study, associations with the derived measure of free testosterone narrowly missed statistical significance.

Alternatively, there could be differential effects of BPA on the metabolism of testosterone and estrogen. A study of steroid hormone production in rat ovarian cells showed that BPA increased both testosterone synthesis and the mRNA expression of steroidogenic enzymes (Zhou et al. 2008). BPA also significantly decreased the activity of enzymes involved in the hydroxylation of testosterone, including the cytochrome P450 isoforms for testosterone 2 α-hydroxylase and testosterone 6 β-hydroxylase, CYP2C11/6 and CYP3A2/1, respectively, in isolated rat livers (Hanioka et al. 1998), both of which could lead to a net increase in circulating testosterone. The possibility that BPA could interfere with the RIA used to quantify serum testosterone is unlikely given the low cross-reactivity shown by the anti-testosterone antibody used in the assay and is further discounted by mathematical modeling studies showing negligible effects of xenoestrogens on the displacement of bound hormone and tracer during binding and extraction steps *in vitro* (Heringa et al. 2004).

It is also plausible that an androgenic environment leads to alterations in the metabolism of BPA, that is, reverse causation. Metabolism of BPA in the intestine and liver catalyzed by uridine diphosphate-glucuronosyl transferase (UGT) yields the major urinary metabolite BPA-glucuronide (Teeguarden et al. 2005). The level of both UGT activity and transcription has been shown to be downregulated by androgens (Guillemette et al. 1997; Takeuchi et al. 2004), which could result in an increase in serum BPA concentration under hyperandrogenic conditions. However, it is unlikely that such metabolic change could alter 24-hr urinary BPA excretion in the context of repeated ingestion of BPA at the population level and the limited increase in testosterone concentrations evident in our analysis.

Urinary BPA concentrations have previously been reported in 100 pregnant European women, with 82% of the study population showing detectable levels of BPA, median concentration 1.2 ng/mL (Ye et al. 2008). This concentration is lower than the mean value presented here, 3.59 ng/mL (95% CI, 3.42–3.77 ng/mL), although there are differences in age and sex profiles. Most studies have reported values from spot urine samples with or without correction for creatinine, with mean concentrations around 3 ng/mL (Dekant and Volkel 2008; Vandenberg et al. 2010) and 95th percentiles in the range of 11.5 ng/mL (Ye et al. 2008) to 16 ng/mL (Calafat et al. 2005). Here, we used 24-hr urine collection to calculate a mean daily excretion rate of 5.63 μg/day (95% CI, 5.67–5.90 μg/day). In an earlier Japanese study, Arakawa et al. (2004) reported median daily urinary excretions of BPA of 1.3–5.0 μg/day, with a maximum daily intake of BPA per body weight of 0.23 μg/kg/day based on 24-hr urine samples collected from 36 men; the median daily uptake was given as 0.02 μg/kg body weight. In controlled, acute human exposure studies, peak urinary concentrations of BPA metabolites were 4,500–6,800 μg/L 6 hr after oral administration of 60–80 μg/kg body weight. Based on these figures and assuming complete and rapid excretion, Dekant and Volkel (2008) suggested that a daily excretion rate of around 5 μg/L, as seen in the general population, indicates ingestion of < 25 μg of BPA in the hours prior to sampling (the maximum daily reference dose is 50 μg/kg/day). However, there are no actual *in vivo* data on the rate at which unconjugated BPA is converted to BPA-glucuronide in humans, only estimates. BPA is lipophilic with a log octanol–water partition coefficient (log *K*_ow_) between 2.2 and 3.82, and it may partition to lipid-rich tissues, a suggestion supported by population-based half-lives for BPA calculated by Stahlhut et al. (2009) to be significantly longer than previous predictions of 6 hr. Given the correlations with BMI and waist circumference seen here, a true estimation of exposure rates remains a priority.

There are limitations to this study that should be borne in mind when interpreting the results. First, replication is required in an independent study population to exclude chance as an explanation, although the small *p*-value in fully adjusted models and the broad consistency with previous work suggest this is unlikely. Second, the analysis is based on a single day of BPA excretion, which is clearly not a perfect measure of longer term exposure given that human health effects are most likely associated with long-term low-dose exposure. However, using the 24-hr urine specimens is likely to be more accurate than previously published work, which has been based on spot urine samples with post hoc adjustment to try to correct for concentration effects. Spot urine samples themselves have been shown to be moderately sensitivity for predicting an individual’s tertile categorization (Mahalingaiah et al. 2008). Misclassification due to this single-day snapshot of excretion will have resulted in a smaller (diluted) estimate of the strength of association between BPA and total testosterone concentrations: the true associations are likely to be much stronger.

Third, the cross-sectional nature of the association reported here needs to be treated with caution. It is also theoretically possible, for example, that those with higher testosterone concentrations alter their diet in such a way as to increase BPA exposure, or, as noted above, that higher testosterone concentrations are themselves responsible for altering metabolism of BPA. It is unclear, however, why altered metabolism would alter our measure of 24-hr excretion systematically, because all BPA is thought to be excreted in the urine in humans sooner or later. We previously reported positive associations between urinary BPA and prevalence of cardiovascular disease (Lang et al. 2008; Melzer et al. 2010). The relationship between circulating testosterone and cardiovascular risk remains to be comprehensively established, although an increased risk of cardiovascular adverse events was recently reportedly in a trial of testosterone supplementation in older men (Basaria et al. 2010).

Future work needs to replicate the association found and to clarify the mechanisms involved. Showing that raised BPA levels precede the increase in testosterone concentrations would establish the temporal sequence of changes and exclude reverse causation. However, a concurrent change in testosterone levels with BPA exposure would remain biologically important. A large-scale exposure trial may be necessary to clarify the association we identified, although the logistics and ethics of such a trial would require careful thought.

## Conclusions

Mean daily exposure to BPA among an Italian adult population sample is in line with previous estimates from the United States, with wide variations around the mean. We found an association between higher daily excretion of BPA and total testosterone concentrations among men. The mechanisms involved in this possible endocrine disruption need clarification.

## Figures and Tables

**Table 1:** Geometric mean (GM) and selected population percentiles of urinary BPA concentrations and daily excretion of the study sample.

			Percentile (95% CI)						
BPA variable	*n* (%)	GM (95% CI)	5th	10th	25th	50th	75th	90th	95th
All									
UER (μg/day)	715	5.63 (5.37–5.90)	2.1 (1.9–2.3)	2.6 (2.5–2.8)	3.7 (3.6–3.9)	5.6 (5.1–5.8)	8.3 (7.7–8.7)	11.8 (10.9–12.7)	16.4 (14.0–20.1)
Urinary concentration (ng/mL)	720	3.59 (3.42–3.77)	1.3 (1.2–1.4)	1.6 (1.5–1.7)	2.3 (2.1–2.4)	3.5 (3.3–3.7)	5.4 (5.0–5.9)	8.0 (7.4–9.5)	11.5 (10.3–13.7)

Sex									
Male									
UER (μg/day)	332 (46.4)	6.26 (5.87–6.68)	2.5 (2.0–2.7)	3.0 (2.6–3.3)	4.3 (3.9–4.6)	6.1 (5.7–6.8)	9.0 (8.3–9.7)	12.5 (11.7–15.4)	16.7 (14.5–23.7)
Urinary concentration (ng/mL)	334 (46.4)	4.02 (3.76–4.31)	1.5 (1.4–1.6)	1.8 (1.6–2.0)	2.4 (2.3–2.7)	3.9 (3.6–4.3)	6.3 (5.7–6.7)	9.8 (8.1–10.9)	13.0 (10.7–14.8)
Female									
UER (μg/day)	383 (53.6)	5.14 (4.81–5.49)	2.0 (1.8–2.2)	2.4 (2.2–2.6)	3.5 (3.2–3.7)	4.9 (4.5–5.3)	7.3 (6.7–8.2)	10.7 (9.9–12.3)	14.4 (12.2–20.4)
Urinary concentration (ng/mL)	386 (53.6)	3.25 (3.04–3.47)	1.1 (1.1–1.3)	1.4 (1.3–1.6)	2.1 (2.0–2.3)	3.2 (2.9–3.4)	4.7 (4.4–5.2)	7.2 (6.5–7.8)	11.0 (7.7–14.1)

Age group (years)									
20–40									
UER (μg/day)	109 (15.2)	6.61 (5.98–7.31)	2.6 (2.3–3.2)	3.2 (2.6–3.8)	4.7 (4.0–5.3)	6.7 (5.8–7.7)	8.9 (8.3–10.9)	12.5 (11.2–16.6)	16.9 (12.6–24.1)
Urinary concentration (ng/mL)	111 (15.4)	4.31 (3.86–4.82)	1.6 (1.2–2.1)	2.1 (1.6–2.3)	3.2 (2.4–3.6)	4.4 (4.0–4.8)	6.0 (5.6–6.8)	8.3 (7.0–12.0)	12.2 (8.4–17.4)
41–65									
UER (μg/day)	157 (22.0)	6.69 (6.04–7.40)	2.7 (2.2–3.2)	3.2 (2.8–3.6)	4.6 (4.0–5.0)	6.2 (5.6–6.9)	9.2 (8.1–10.3)	16.1 (11.3–21.3)	23.8 (16.7–40.7)
Urinary concentration (ng/mL)	157 (21.8)	3.95 (3.53–4.42)	1.4 (1.2–1.5)	1.5 (1.4–2.0)	2.4 (2.1–2.8)	3.7 (3.3–4.4)	5.8 (5.1–6.7)	9.6 (7.5–15.3)	16.7 (11.1–22.3)
66–74									
UER (μg/day)	449 (62.8)	5.10 (4.80–5.41)	1.9 (1.6–2.1)	2.4 (2.1–2.6)	3.5 (3.1–3.6)	4.9 (4.5–5.3)	7.4 (7.0–8.3)	10.9 (9.9–12.1)	14.2 (12.2–17.1)
Urinary concentration (ng/mL)	452 (62.8)	3.32 (3.12–3.53)	1.2 (1.1–1.3)	1.5 (1.3–1.6)	2.1 (2.0–2.3)	3.2 (2.9–3.4)	4.8 (4.4–5.6)	7.6 (7.0–8.9)	10.7 (9.3–12.8)

**Table 2:** Geometric means (GMs) of BPA excretion, by covariate status, plus age, sex, and study site using adjusted regression estimates of association.

Variable	*n* (%)	GM (μg/day) (95% CI)	*p*-Value^a^
Education (years)			
0	3 (0.4)	4.72 (1.80 to 12.35)	(dropped)
1–5	364 (50.9)	5.06 (4.75 to 5.39)	—b
6–8	152 (21.3)	6.27 (5.59 to 7.02)	0.071
9–13	123 (17.2)	6.07 (5.46 to 6.75)	0.968
14–19	63 (8.8)	6.99 (5.99 to 8.17)	0.170
≥ 20	10 (1.4)	5.84 (3.82 to 8.94)	0.576

BMI category (kg/m^2^)			
Underweight (BMI 0–18.5)	4 (0.6)	2.74 (1.29 to 5.81)	(dropped)
Normal (BMI 18.5–25)	215 (30.1)	5.67 (5.22 to 6.16)	—b
Overweight (BMI 25–30)	314 (43.9)	5.84 (5.43 to 6.27)	0.296
Obese I (BMI 30.1–34.9)	138 (19.3)	5.66 (5.04 to 6.34)	0.369
Obese II (BMI ≥ 35)	32 (4.5)	4.85 (3.94 to 5.98)	0.738
Unknown	12 (1.6)	3.46 (2.65 to 4.52)	(dropped)

Smoking history			
Never	380 (53.2)	3.20 (3.03 to 3.37)	—b
Former	171 (23.9)	3.76 (3.50 to 4.05)	0.259
Current	164 (22.9)	3.95 (3.62 to 4.32)	0.773

Continuous measures			
Waist circumference (cm)	715	β = 0.0062 (0.0016 to 0.0108)	0.013
Weight (kg)	715	β = 0.0064 (0.0023 to 0.0104)	0.003
Urinary creatinine concentration (mg/dL)	715	β = −0.0012 (–0.0025 to 0.0003)	0.116

a Adjusted for age, sex, and site.

b The base category against which the others are tested.

**Table 3:** Simple and fully adjusted regression models of the associations between BPA (μg/day) and 17β-estradiol and testosterone concentrations for men.

			Age and study-site adjusted		Fully adjusted^a^	
Hormone	*n*	Geometric mean (95% CI)	β-Coefficient (95% CI)	*p*-Value	β-Coefficient (95% CI)	*p*-Value
17β-Estradiol (pg/mL)	293	12.89 (12.26 to 13.56)	−0.00004 (−0.0086 to 0.0085)	0.992	0.0002 (−0.011 to 0.011)	0.975
Total testosterone (ng/mL)	307	4.55 (4.42 to 4.69)	0.0237 (0.0006 to 0.0468)	0.044	0.046 (0.015 to 0.076)	0.004
SHBG (nmol/mL)	316	80.84 (76.60 to 85.30)	−0.0009 (−0.0095 to 0.0076)	0.830	0.0011 (−0.0075 to 0.0096)	0.805
Free testosterone (ng/dL)	316	4.72 (4.50 to 4.95)	0.0081 (−0.0012 to 0.0175)	0.089	0.0088 (−0.0009 to 0.0185)	0.075

a Full models were adjusted for age, study site, smoking, BMI, weight, waist, and urinary creatinine concentration.

**Table 4:** Simple and fully adjusted regression models of the associations between BPA (μg/day) and 17β-estradiol, testosterone, and SHBG concentrations for women.

			Age and study-site adjusted		Fully adjusted^a^	
Hormone	*n*	Geometric mean (95% CI)	β-Coefficient (95% CI)	*p*-Value	β-Coefficient (95% CI)	*p*-Value
Premenopause						
17β-Estradiol (pg/mL)	57	22.4 (16.7–30.0)	−0.026 (−0.066 to 0.014)	0.204	−0.022 (−0.066 to 0.0229)	0.325
Total testosterone (ng/mL)	61	0.69 (0.61–0.77)	−0.004 (−0.015 to 0.007)	0.451	−0.007 (−0.018 to 0.004)	0.192
SHBG (nmol/mL)	60	134.3 (111.6–161.5)	0.029 (0.004 to 0.054)	0.024	0.038 (0.013 to 0.063)	0.004
Postmenopause						
17β-Estradiol (pg/mL)	290	5.3 (4.8–5.7)	−0.002 (−0.010 to 0.005)	0.516	−0.003 (−0.010 to 0.005)	0.448
Total testosterone (ng/mL)	294	0.54 (0.49–0.59)	−0.0003 (−0.0036 to 0.0030)	0.871	−0.001 (−0.004 to 0.002)	0.555
SHBG (nmol/mL)	299	105.2 (98.8–112.1)	0.002 (−0.004 to 0.008)	0.541	0.003 (−0.003 to 0.009)	0.272

a Full models adjusted for age, study site, smoking, BMI, weight, waist, and urinary creatinine concentration.
